# Schisandrin B Targets the PPARγ-MAPK Signaling Axis to Ameliorate High-Fat MCD Diet-Induced MASLD in Mice

**DOI:** 10.3390/ph19091367

**Published:** 2026-08-28

**Authors:** Xi-Yuan Feng, Meng Gao, Fei-Long Liu, Ming-Ze Li, Xiao-Li Cui, Meng-Yang Wang, Zhi-Hong Zhang, He Li, Chun-Mei Wang, Jing-Hui Sun

**Affiliations:** School of Pharmacy, Beihua University, Jilin 132013, China; 18004369287@163.com (X.-Y.F.); s9912062636@163.com (M.G.); m18831442942@163.com (F.-L.L.); 13011701698@163.com (M.-Z.L.); cuixl@beihua.edu.cn (X.-L.C.); 15643584357@163.com (M.-Y.W.); zhzhang518@163.com (Z.-H.Z.); yitonglh@126.com (H.L.); wangcm74@126.com (C.-M.W.)

**Keywords:** Schisandrin B, peroxisome proliferator-activated receptor gamma, metabolic dysfunction-associated steatotic liver disease, lipid metabolism, fibrosis

## Abstract

**Objectives:** This study focuses on exploring the mechanism by which Schisandrin B (Sch B) regulates metabolic dysfunction-associated steatotic liver disease (MASLD) mice induced by a high-fat methionine–choline-deficient (MCD) diet through the activation of peroxisome proliferator-activated receptor γ (PPARγ). **Methods**: Male C57BL/6 mice were fed a high-fat MCD diet for 8 weeks to establish a mouse MASLD model, and the effects of Sch B on MASLD and the mechanisms were investigated. PPARγ overexpression (OE) was induced by adeno-associated virus (AAV) administration via intrahepatic portal vein injection in mice, and a negative control (NC-OE) was also established. Body weight; wet liver weight; hepatic index; serum levels of alanine aminotransferase (ALT), aspartate aminotransferase (AST), tumor necrosis factor-α (TNF-α), interleukin-6 (IL-6), and interleukin-1β (IL-1β); and hepatic triglyceride (TG) levels were measured in the mice. The histopathology and lipid deposition were observed by hematoxylin and eosin (H&E) staining and Oil Red O staining, while the fibrosis was assessed using Masson staining. Western blot was employed to detect the expression levels of PPARγ, sterol regulatory element-binding protein 1c (SREBP-1c), carnitine palmitoyltransferase 1A (CPT1A), transforming growth factor β1 (TGF-β1), α-smooth muscle actin (α-SMA), collagen type I (collagen I), Smad family members 2/3 (Smad2/3), c-Jun N-terminal kinase (JNK), p38 mitogen-activated protein kinase (p38), and extracellular signal-regulated kinase 1/2 (ERK1/2), along with the phosphorylation activation status of these kinases. **Results**: It was confirmed that Sch B caused effects similar to those induced by PPARγ overexpression, reducing the hepatic index, AST, and ALT levels while alleviating lipid accumulation and fibrosis; and upregulating PPARγ and CPT1A while inhibiting SREBP-1c; and the phosphorylation of the TGF-β/Smad and MAPK pathways were involved in the mechanisms. **Conclusions**: Sch B can alleviate high-fat MCD-induced MASLD by activating PPARγ in mice.

## 1. Introduction

The global prevalence of metabolic dysfunction-associated steatotic liver disease (MASLD) has surpassed that of other chronic liver diseases, and it encompasses a complete spectrum ranging from simple steatosis to metabolic dysfunction-associated steatohepatitis (MASH), liver fibrosis, cirrhosis, and even hepatocellular carcinoma, imposing increasingly severe public health burdens [1]. According to the “multiple hits” hypothesis, the pathogenesis of MASLD is not driven by a single factor, and the lipotoxic effects resulting from excessive intracellular lipid accumulation are considered the initiating trigger of the entire cascade among the multiple factors. The prolonged lipotoxicity may disrupt hepatic redox homeostasis and induce a chronic inflammatory microenvironment to further stimulate hepatic stellate cells to transition from a quiescent state to an activated phenotype, and the activated stellate cells then produce large amounts of extracellular matrix and primarily collagen, driving progressive fibrosis [2,3,4]. It follows that there may be a close pathological interplay between lipotoxicity and fibrosis, and the interactive progression of both may be the core mechanism of MASLD progressing to end-stage liver diseases.

Peroxisome proliferator-activated receptor gamma (PPARγ), a member of the nuclear receptor superfamily, plays a central role in lipid metabolism homeostasis and insulin sensitivity regulation, and its critical roles in suppressing inflammation and fibrosis have been further revealed in recent studies [5]. PPARγ exhibits dual functionality in the intrahepatic microenvironment: at the hepatocyte level, it finely regulates fatty acid synthesis and catabolism; while in hepatic stellate cells, its activation effectively maintains the quiescent phenotype and prevents their transdifferentiation into myofibroblasts with pro-fibrotic properties [6].

In terms of the molecular mechanism, the transcriptional regulation function of PPARγ is mainly achieved through two pathways: one is to form a heterodimer with retinoic acid X receptor (RXR) and then bind to the PPAR response element (PPRE) of the target gene promoter, directly initiating the transcription of metabolic genes such as fatty acid oxidation; the second is to interfere with the binding of transcription factors such as AP-1 and NF-κB to DNA through the trans-inhibitory mechanism, thereby blocking the expression of inflammation and fibrosis-related genes without directly binding to target genes [7]. Once PPARγ expression is downregulated or its activities are impaired, hepatic stellate cells are more easily activated, initiating and accelerating excessive deposition of extracellular matrix [8]. In parallel, the MAPK signaling cascade (including JNK, p38, and ERK1/2) also plays an important regulatory role in the process of liver inflammation and fibrosis [9]. For this reason, PPARγ is considered an important potential intervention node for blocking the vicious cycle of inflammation to fibrosis in MASLD [10].

As *Schisandra chinensis* a traditional medicinal and edible Chinese herb, its active component Schisandrin B (Sch B) has been proven to possess multiple pharmacological activities and shows potential application value in the treatment of various liver diseases. In our previous network pharmacology screening, molecular docking analysis, and in vitro and in vivo experimental verification, PPARγ was identified as a key target for Sch B to improve liver lipid metabolism disorders in MASLD, and the lipid-lowering effect and intestinal microbiota regulation of Sch B was dependent on PPARγ [11,12,13].

On this basis, a liver-specific PPARγ-overexpression group and a high-dose Sch B intervention group were simultaneously set in this study, aiming to test the following scientific hypotheses: Sch B could activate the PPARγ signaling pathway, on the one hand, to inhibit the excessive accumulation of liver cell lipids and the inflammatory cascade reaction triggered by it, and on the other hand, to block the activation of hepatic stellate cells mediated by the TGF-β/Smad pathway; at the same time, Sch B could also exert a multi-level protective effect in a high-fat MCD diet-induced MASLD mouse model by regulating the phosphorylation cascade of the MAPK signaling axis, synergistically inhibiting the release of pro-inflammatory cytokines and excessive deposition of extracellular matrix.

## 2. Results

### 2.1. Downregulation of PPARγ-Expression Levels in the Liver Tissue of MASLD Mice

**Figure 1:**
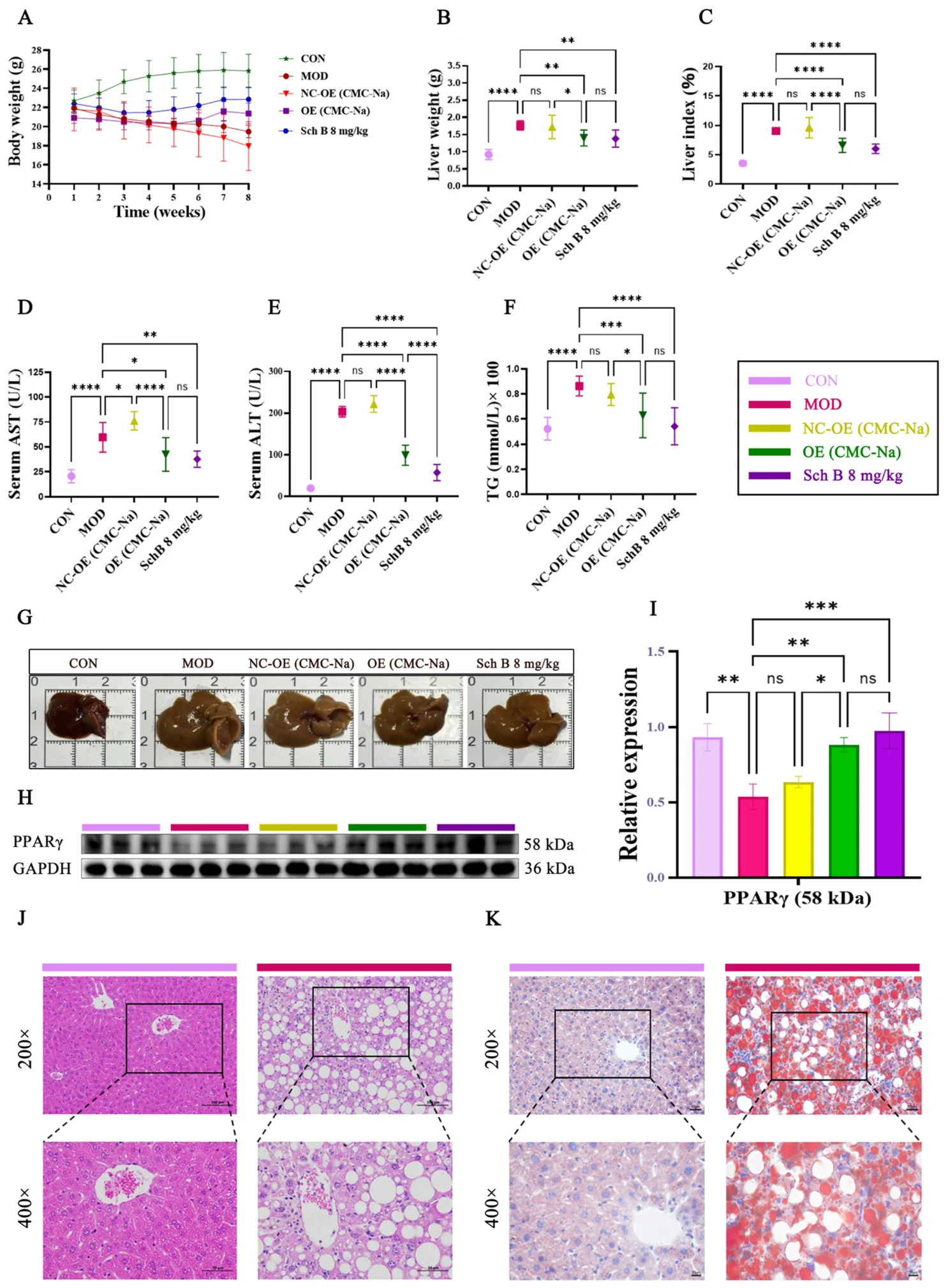
Downregulation of PPARγ-expression levels in the liver tissue of MASLD mice. (**A**) Mouse weight; (**B**) wet liver weight; (**C**) liver index; (**D**) AST levels; (**E**) ALT levels; (**F**) TG levels (mean ± S.E., *n* = 10). (**G**) Volume of isolated liver; (**H**) Western blot of PPARγ; (**J**) liver HE staining; (**K**) liver Oil Red O staining; (**I**) Western blot protein-expression results (mean ± S.E., *n* = 3). ns > 0.05, * *p* < 0.05, ** *p* < 0.01, *** *p* < 0.001, **** *p* < 0.0001.

There were significant differences in the body weight, liver wet weight, liver index, and volume of isolated liver of mice between the CON and MOD groups (Figure 1A–C,G). The HE and Oil Red O staining results of the liver sections showed that the hepatic steatosis, lipid droplet deposition, and inflammatory infiltration of mice in the MOD group were more severe (Figure 1J,K), and the levels of AST and ALT in serum, and TG in liver tissue in MOD-group mice were all elevated (Figure 1D–F) compared to the CON group, indicating that the MASLD model was successfully established. In order to investigate the role of PPARγ in MASLD, the expression level of PPARγ in MASLD was first evaluated. The Western blot results showed that compared with those in the CON group, the expression of PPARγ in the liver tissue of mice in the MOD group was downregulated (Figure 1H,I), suggesting that the downregulation of PPARγ expression may potentially play a role in MASLD.

### 2.2. Effects of Elevated PPARγ Expression on the Lipid Accumulation in MASLD Mice

**Figure 2:**
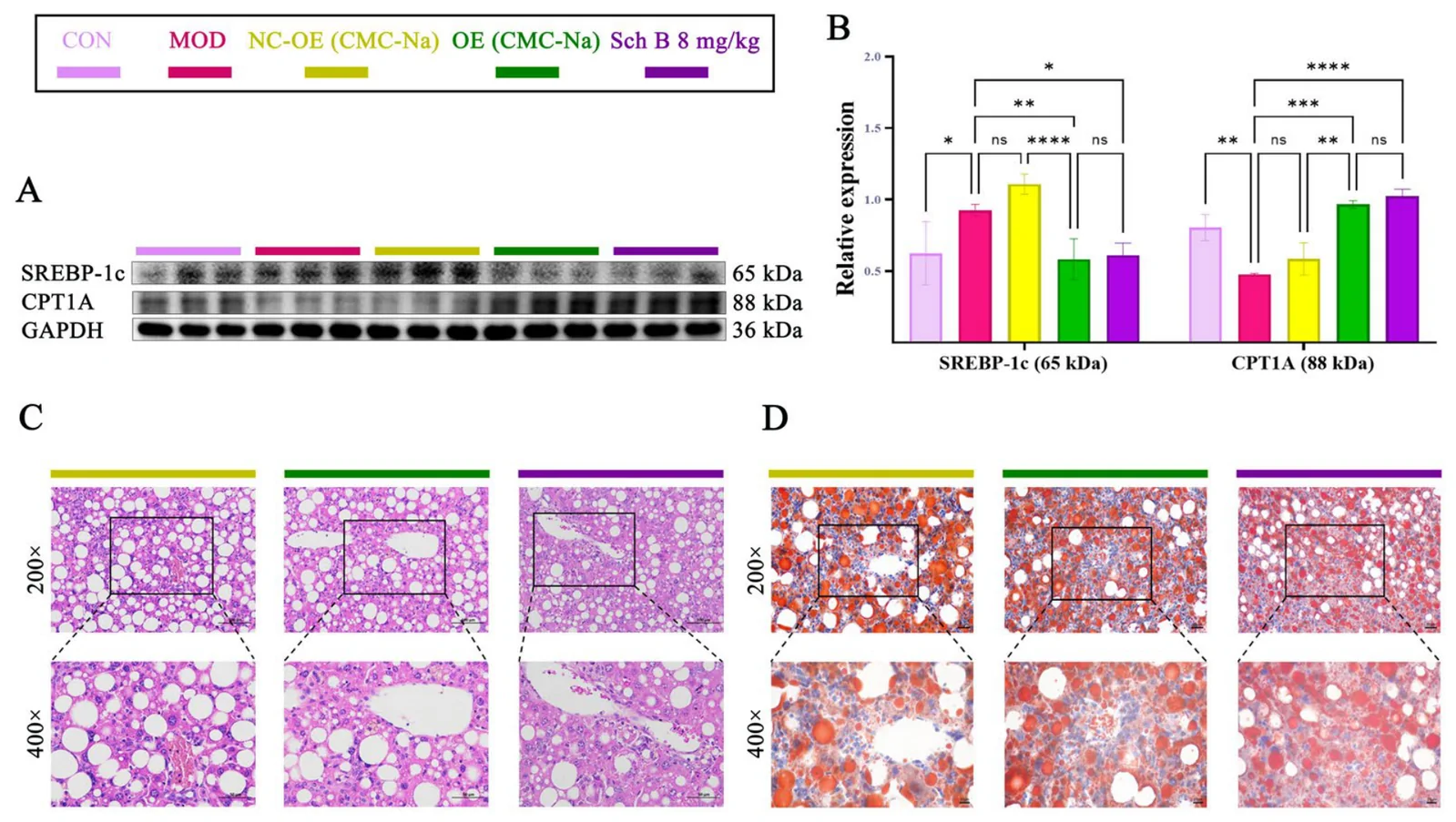
Effects of elevated expression of PPARγ on the lipid accumulation in MASLD mice. (**A**) Western blot of SREBP-1c and CPT1A; (**B**) Western blot protein-expression results; (**C**) liver HE staining; (**D**) liver Oil Red O staining (mean ± S.E., *n* = 3). ns > 0.05, * *p* < 0.05, ** *p* < 0.01, *** *p* < 0.001, **** *p* < 0.0001.

The Western blot results showed that, compared with the MOD group, the expression of PPARγ in the liver tissue was upregulated in the OE (CMC-Na) and Sch B 8 mg/kg groups, with no significant difference between these two groups. In contrast, the NC-OE (CMC-Na) group showed no significant difference compared with the MOD group (Figure 1H,I), and the body weight, wet liver weight, liver index, serum AST, serum ALT, and TG in liver tissue were all decreased (Figure 1A–G). The HE and Oil Red O staining results showed that the lipid deposition and steatosis in the liver tissue of mice in the OE (CMC-Na) group and Sch B 8 mg/kg group were alleviated (Figure 2C,D). The Western blot results showed that, compared with the MOD group, in both the OE (CMC-Na) and Sch B 8 mg/kg groups, the protein expression of SREBP-1c was downregulated, while that of CPT1A was upregulated, with no significant difference between these two treatment groups (Figure 2A,B). In summary, an increase in PPARγ can alleviate the lipid accumulation in MASLD-model mice.

### 2.3. Effects of Elevated PPARγ Expression on the Inflammatory Response in MASLD Mice

**Figure 3:**
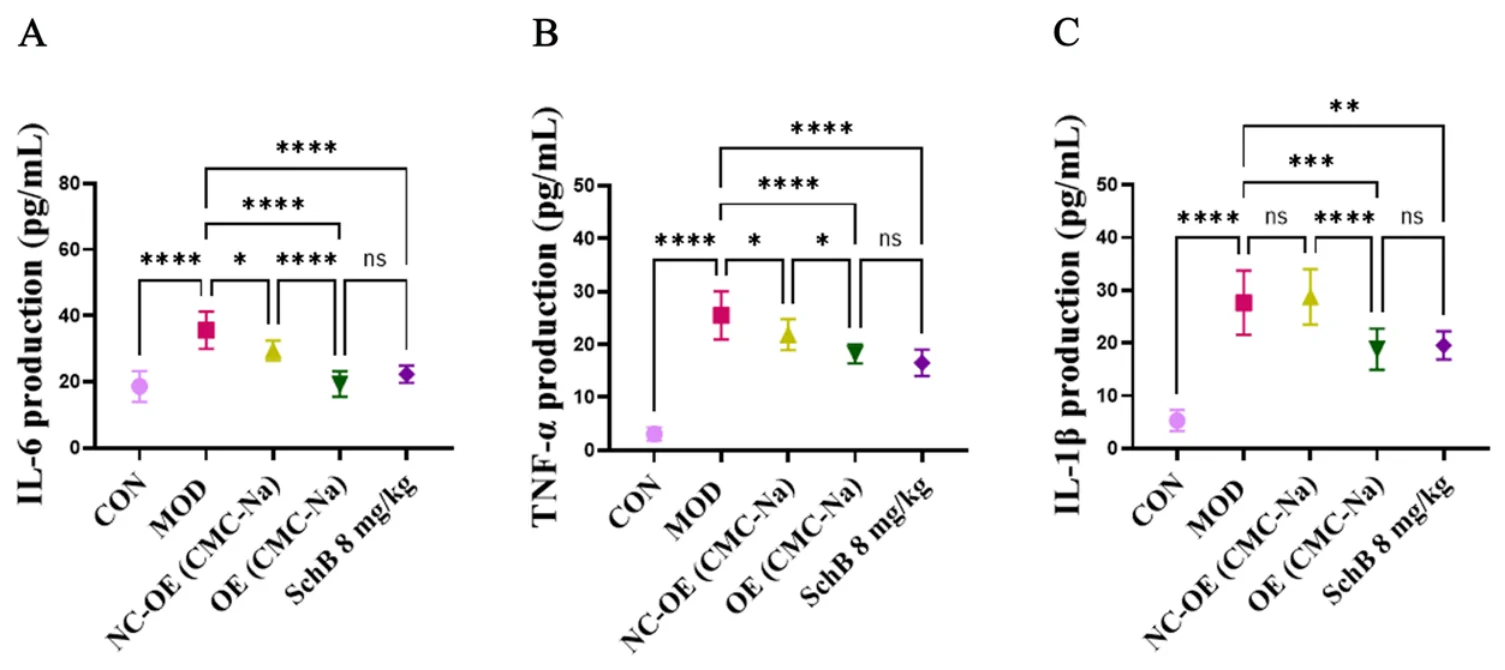
Effects of elevated expression of PPARγ on the inflammatory response in MASLD mice. (**A**) IL-6; (**B**) TNF-α; (**C**) IL-1β (mean ± S.E., *n* = 10). ns > 0.05, * *p* < 0.05, ** *p* < 0.01, *** *p* < 0.001, **** *p* < 0.0001.

The ELISA results showed that compared with those in the MOD group, the levels of inflammatory factors such as TNF-α, IL-6, and IL-1β in the serum of mice in the OE (CMC-Na) and Sch B 8 mg/kg groups were significantly reduced, with no significant difference between these two groups (Figure 3A–C). Furthermore, the liver tissue HE staining results showed that the infiltration degree of inflammatory cells in the liver tissue of mice in the two groups was significantly reduced (Figure 2C). The above results suggest that upregulating PPARγ expression can significantly alleviate the liver inflammatory response and inflammatory infiltration in MASLD mice.

### 2.4. Effects of Elevated PPARγ Expression on the Liver Fibrosis in MASLD Mice

**Figure 4:**
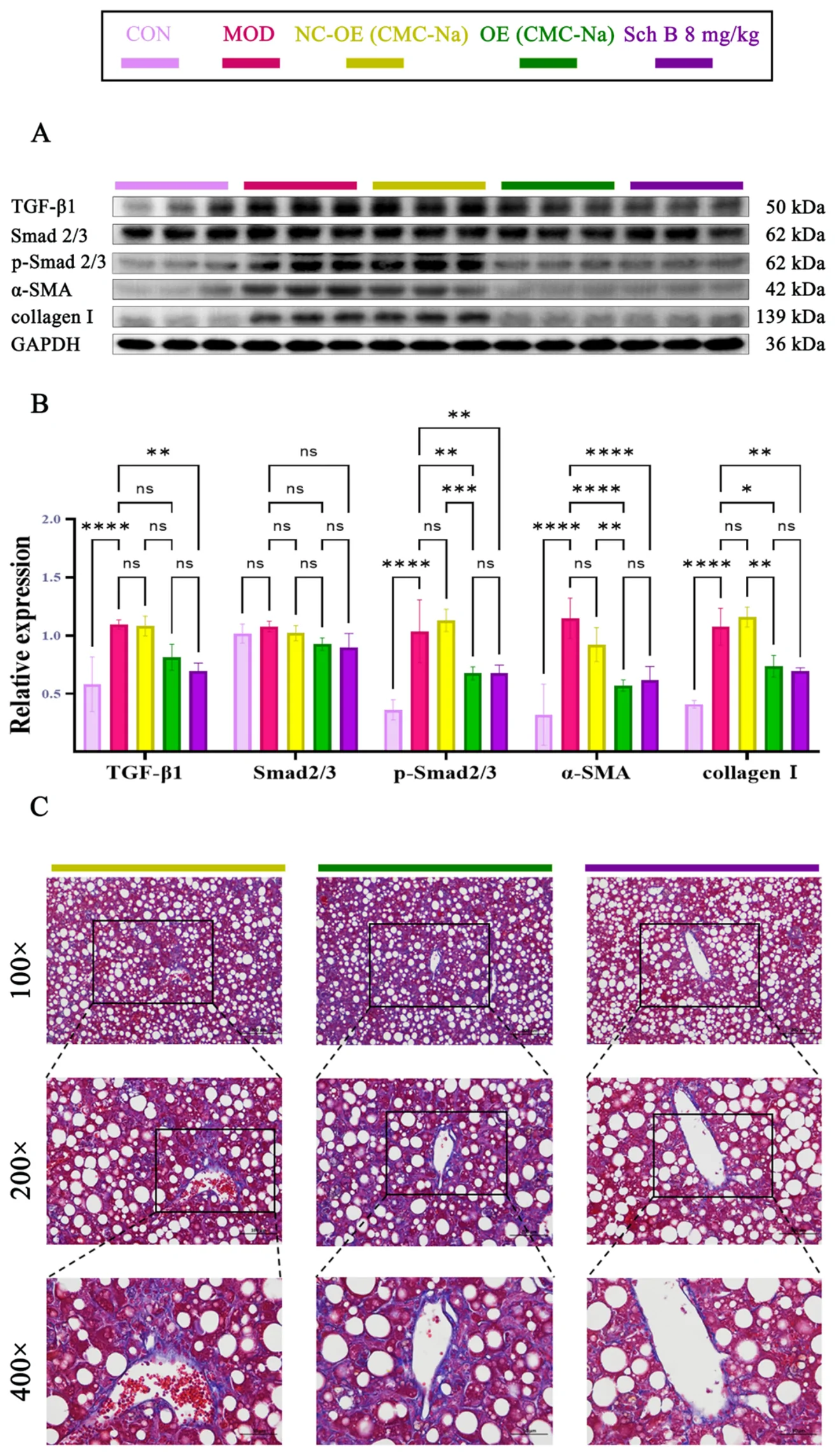
Effects of elevated PPARγ expression on the liver fibrosis in MASLD mice. (**A**) Western blot of TGF-β1, α-SMA, collagen I, Smad2/3, and p-Smad2/3; (**B**) Western blot protein-expression results; (**C**) Masson staining of liver (mean ± S.E., *n* = 3). ns > 0.05, * *p* < 0.05, ** *p* < 0.01, *** *p* < 0.001, **** *p* < 0.0001.

The Masson staining results showed that there was a large proliferation of collagen fibers in the liver tissue of mice in NC-OE (CMC-Na) group, and compared with that in NC-OE (CMC-Na) group, the collagen deposition in the liver tissue of mice in the OE (CMC-Na) group and Sch B 8 mg/kg group was significantly alleviated, with a narrower fibrous septa and significant improvement in the degree of liver fibrosis (Figure 4C). The Western blot detection further confirmed that the expression levels of fibrosis-related proteins such as TGF-β1, α-SMA, collagen I, and p-Smad2/3 were significantly downregulated in the liver tissues of mice in the two groups mentioned above, with no significant difference between these two groups (Figure 4A,B). Therefore, it can be speculated that upregulating PPARγ can significantly alleviate the progression of liver fibrosis in MASLD mice.

### 2.5. Effects of PPARγ on the Progression of MASLD by Regulating the MAPK Signaling Pathway

**Figure 5:**
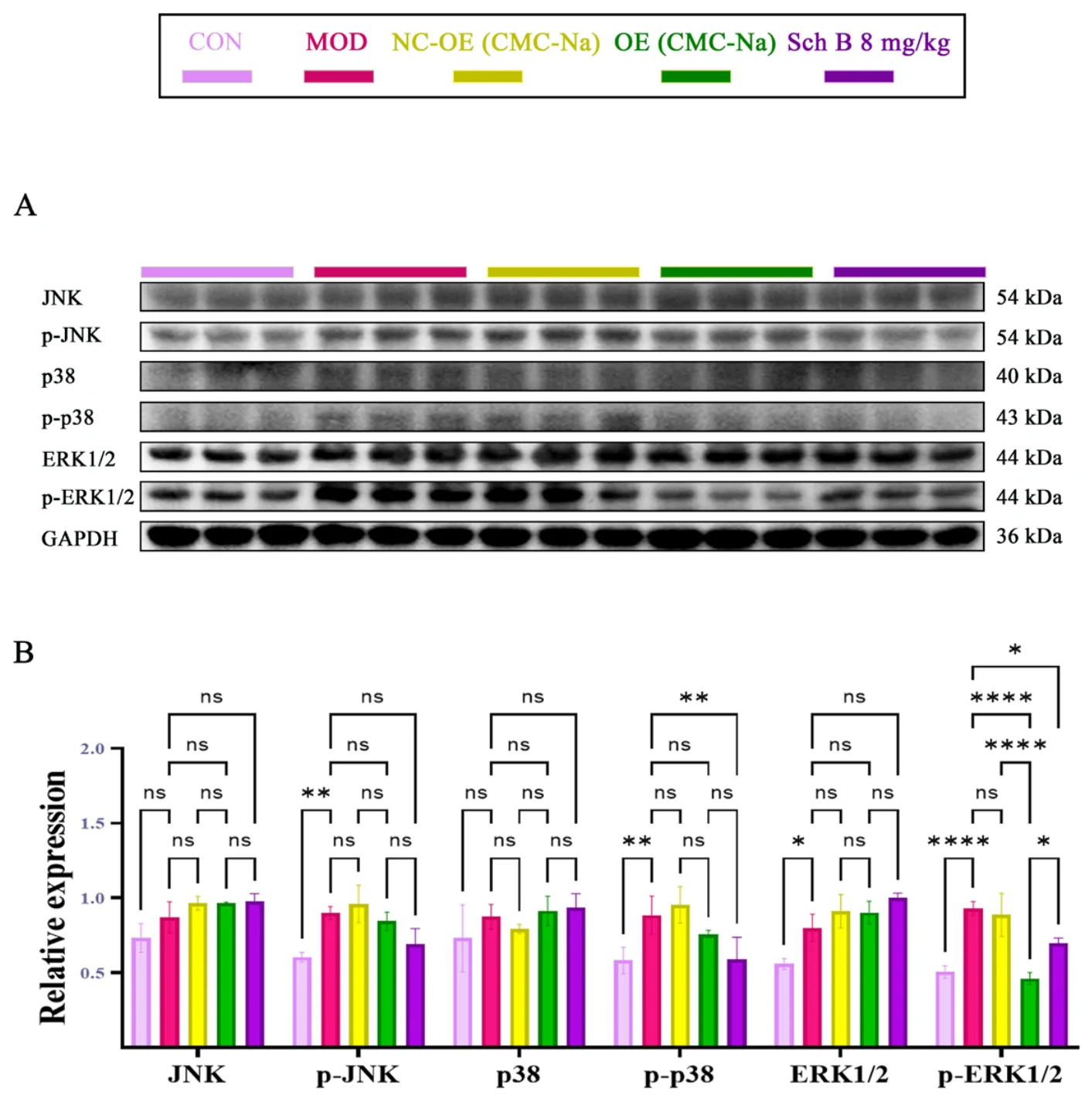
Effects of PPARγ on the progression of MASLD by regulating the MAPK signaling pathway. (**A**) Western blot of JNK, p-JNK, p38, p-p38, ERK1/2, and p-ERK1/2; (**B**) Western blot protein-expression results (mean ± S.E., *n* = 3). ns > 0.05, * *p* < 0.05, ** *p* < 0.01, **** *p* < 0.0001.

Based on the above experimental results, it could be concluded that upregulating PPARγ could significantly improve lipid metabolism disorders, inflammatory reactions, and liver fibrosis in MASLD mice. To further clarify the regulatory mechanism, we focused on exploring the regulatory effect of PPARγ on the MAPK signaling pathway and its mediating role in PPARγ intervention in the progression of MASLD, so the total protein-expression and phosphorylation-activation levels of key molecules (JNK, p38, and ERK1/2) in the MAPK signaling pathway in the liver tissues of mice were further detected by Western blot (Figure 5A,B). The results showed that compared with those in the CON group, the expression levels of the p-JNK, p-p38, and p-ERK1/2 proteins in the liver tissue of mice in the MOD group were significantly upregulated; compared with those in the MOD group, the expression levels of the p-JNK, p-p38, and p-ERK1/2 proteins in the liver tissues of mice in the OE (CMC-Na) and Sch B 8 mg/kg groups were significantly downregulated, with no significant difference between these two groups; in addition, there were no significant differences in the expression levels of p-JNK, p-p38, p-ERK1/2, and total protein in the liver tissue between the NC-OE (CMC-Na) group and MOD group, ruling out the influence of AAV vector and solvent CMC-Na on the MAPK signaling pathway, which demonstrates the reliability of the experimental results.

In summary, PPARγ can inhibit the phosphorylation activation of key kinases in the MAPK signaling pathway to block the abnormal activation of the MAPK signaling pathway, and thereby alleviate liver lipid accumulation, inflammatory infiltration, and fibrosis in MASLD mice, suggesting that the MAPK signaling pathway may be an important downstream target regulated by PPARγ for the progression of MASLD, and Sch B can exert a protective effect on MASLD by activating PPARγ-mediated inhibition of the MAPK signaling pathway.

## 3. Discussion

Diet-induced animal models are an important method for simulating the pathological process of human MASLD. A high-fat methionine–choline-deficiency (MCD) feed used in this study was improved based on the classic MCD formula, in which while retaining the methionine–choline deficiency and the damage to the very low-density lipoprotein secretion in the liver, the fat–energy supply ratio was increased to about 60% [14]. This improvement not only significantly exacerbated the hepatic steatosis, but also more stably and rapidly induced the hepatic inflammatory infiltration and perisinusoidal fibrosis, which was considered closer to the pathological state of metabolic disorders in clinical MASLD patients [15]. In addition, to eliminate the potential influence of gender and age on the experimental results, male C57BL/6J mice aged 6–8 weeks were used in this study. Studies have shown that there are significant gender differences in liver injury in diet-induced MASLD/MASH mouse models, with male mice exhibiting more severe lipid degeneration, inflammation, fibrosis, and liver cell death than female mice [16,17]. Therefore, selecting male mice can help obtain a more significant and uniform damage model phenotype, thereby objectively evaluating the actual effect of drug intervention. Schisandrin B (Sch B), a major active constituent of *Schisandra chinensis*, has garnered extensive attention for its protective roles in metabolic liver diseases. We adopted the 8 mg/kg dose according to our prior published findings, which confirmed its safety and ability to produce stable hepatoprotective effects in mouse models [12]. 

At present, the high incidence rate of MASLD has become a public health problem that cannot be ignored. The disease starts with a large accumulation of lipids in the liver, gradually goes through chronic inflammation and fibrosis stages, and can eventually progress to cirrhosis and hepatocellular carcinoma [1]. Although abnormal lipid metabolism and insulin resistance are considered the core pathogenesis, drugs that can directly reverse or block the pathological process are still extremely limited [3]. Even resmetirom, approved by the FDA under accelerated review in 2024, only achieved histological relief in some patients during its critical phase III clinical trial, while also exhibiting some adverse reactions such as itching, elevated cholesterol, and risk of cardiovascular events [18]. In this context, revealing new molecular regulatory nodes and developing effective intervention strategies based on them is an urgent breakthrough direction in this field. Based on this, a high-fat MCD feed was selected to construct a mouse MASLD model, and two strategies of liver PPARγ overexpression and Sch B administration in parallel were adopted in this experiment, aiming to systematically elucidate the functional role of PPARγ in MASLD and the regulatory effect of Sch B. The pivotal regulatory role of the PPARγ-MAPK signaling pathway in the progression of MASLD was confirmed in this study, which may provide some ideas for new candidate targets and mechanisms for exploring drug treatment options for MASLD.

Serum AST and ALT are core biochemical indicators reflecting liver cell damage, and their elevation usually indicates impaired integrity of the liver cell membrane or cell necrosis [19]. In this study, AST and ALT levels in the serum of mice in the MOD group were significantly elevated, accompanied by changes in body weight, large deposition of lipids in the liver, and typical histological features such as steatosis, inflammatory infiltration, and fibrosis. The simultaneous presence of these pathological features can directly confirm the successful establishment of the MASLD model. At the molecular level, the suppression of PPARγ expression is considered a key link between lipid metabolism disorders and liver injury: when PPARγ activity is insufficient, the fatty-acid-uptake and de novo-synthesis pathways in liver cells become out of control, while mitochondrial β-oxidation capacity decreases, and the dual imbalance of lipid clearance disorders and synthesis may exacerbate the problem, ultimately driving liver steatosis and promoting subsequent inflammation and fibrosis [20]. The experimental data showed that the AAV-mediated overexpression of PPARγ and the treatment with Sch B 8 mg/kg could significantly increase PPARγ levels in the liver tissue and alleviate the liver injury, and the effects in both groups were comparable, while there was no significant difference in the various indicators between the NC-OE (CMC-Na) group and MOD group, confirming the target specificity of the AAV intervention. To sum up, the effect of Sch B on ameliorating liver injury can be attributed to its upregulation or functional activation of PPARγ, which is consistent with previous mechanistic hypotheses, further clarifying that PPARγ is a functional target of Sch B in ameliorating MASLD.

Our previous KEGG pathway enrichment analysis showed that the MAPK (mitogen-activated protein kinase) signaling cascade was one of the pathways significantly enriched in MASLD-related targets [12], suggesting that the abnormal activation of this pathway may be involved in the progression of MASLD. For this purpose, based on confirming PPARγ as the core target, the phosphorylation activation levels of three key branch kinases of the MAPK pathway, including JNK, p38, and ERK1/2, were further detected in this study. The MAPK signaling pathway is the core signaling network for cells to respond to external stress, inflammation, and metabolic disorders. Its downstream JNK mainly drives inflammation and lipid toxicity damage, p38 is closely related to fibrosis process, and ERK1/2 is deeply involved in the metabolic regulation of liver cells [9,21]. All three require phosphorylation (to form p-JNK, p-p38, and p-ERK1/2, respectively) to be active. The Western blot results of this experiment showed that the levels of p-JNK, p-p38, and p-ERK1/2 were significantly upregulated in the liver tissue of mice in the MOD group, and the levels of the phosphorylated proteins mentioned above in both the OE (CMC-Na) group and the Sch B 8 mg/kg group were significantly downregulated, while there was no such effect in the NC-OE (CMC-Na) group, indicating that the activation of PPARγ can negatively regulate the abnormal overactivation of the MAPK signaling pathway by inhibiting the phosphorylation levels of JNK, p38, and ERK1/2, thereby improving lipid accumulation, inflammation, and fibrosis in MASLD, which may be one of the core mechanisms by which PPARγ exerts its liver-protective effects.

At the transcriptional regulatory level of lipid metabolism, SREBP-1c, as a core transcription factor driving fatty acid synthesis, overactivation may accelerate hepatic lipid synthesis and exacerbate lipid accumulation [22]. CPT1A, located on the outer membrane of mitochondria, is a key rate-limiting enzyme for long-chain fatty acids to enter mitochondria for β-oxidation, and the inhibition of its expression directly leads to the inhibition of fatty acid oxidation to further exacerbate liver lipid load [23]. The results of this study showed that the TG content in liver tissue was significantly reduced and the lipid deposition was alleviated in both the OE (CMC-Na) group and Sch B 8 mg/kg group; it is believed that the molecular basis of this can be attributed to the downregulation of the SREBP-1c protein level and the upregulation of CPT1A expression, which synchronously inhibits lipid synthesis and promotes fatty acid oxidation, thereby restoring liver cell lipid homeostasis. In summary, PPARγ may exert a protective effect against lipid metabolism disorders by reversing the upregulation of SREBP-1c and downregulation of CPT1A in MASLD, and Sch B can drive this bidirectional regulatory mechanism by activating PPARγ.

In the inflammatory microenvironment of MASLD, TNF-α acts as an upstream pro-inflammatory factor to drive the initiation and amplification of inflammatory responses, and can inhibit fatty acid β-oxidation, exacerbating lipid accumulation, IL-6 promotes inflammatory cell infiltration and participates in the regulation of lipid metabolism disorders, and IL-1β is mainly responsible for initiating and enhancing the inflammatory cascade while inducing hepatocyte apoptosis and promoting fibrosis progression [24]. It was observed in this experiment that the levels of TNF-α, IL-6, and IL-1β in the serum of mice in the MOD group were significantly increased, and the inflammatory infiltration in the liver tissue was obvious, indicating chronic inflammation; the levels of the aforementioned inflammatory factors were significantly reduced and the liver inflammation was alleviated in both the OE (CMC-Na) group and Sch B 8 mg/kg group. The mechanism can be attributed to the inhibition of MAPK signaling pathway phosphorylation by activating PPARγ, thereby blocking the transcription and release of pro-inflammatory factors to play an anti-inflammatory role.

TGF-β1, a core cytokine that drives the progression of liver fibrosis, can bind to its receptors and then phosphorylate Smad2/3 (forming p-Smad2/3) to enter the nucleus to regulate the transcription of fibrosis-related genes, including the upregulation of the hepatic stellate cell activation marker α-SMA and expression of the major extracellular matrix component collagen I, ultimately leading to massive collagen deposition [4,25,26]. In this study, significant collagen deposition was observed in the liver tissue of mice, and the levels of TGF-β1, α-SMA, collagen I, and p-Smad2/3 proteins were significantly upregulated in the MOD group, confirming the formation of fibrosis; the collagen deposition was alleviated, the expression of the fibrosis markers mentioned above was downregulated, and the phosphorylation of Smad2/3 was reduced significantly in both the OE (CMC-Na) group and Sch B 8 mg/kg group, suggesting that PPARγ may synergistically inhibit liver fibrosis through a dual mechanism. On the one hand, it may directly inhibit the expression of TGF-β1 to reduce hepatic stellate cell activation; on the other hand, it may indirectly block the transmission of TGF-β/Smad signaling by inhibiting the phosphorylation of the MAPK pathway; both work together to inhibit the excessive deposition of extracellular matrix. The overexpression of PPARγ and the intervention of Sch B showed a similar effect, further supporting the anti-fibrotic effect of Sch B by activating PPARγ.

**Figure 6:**
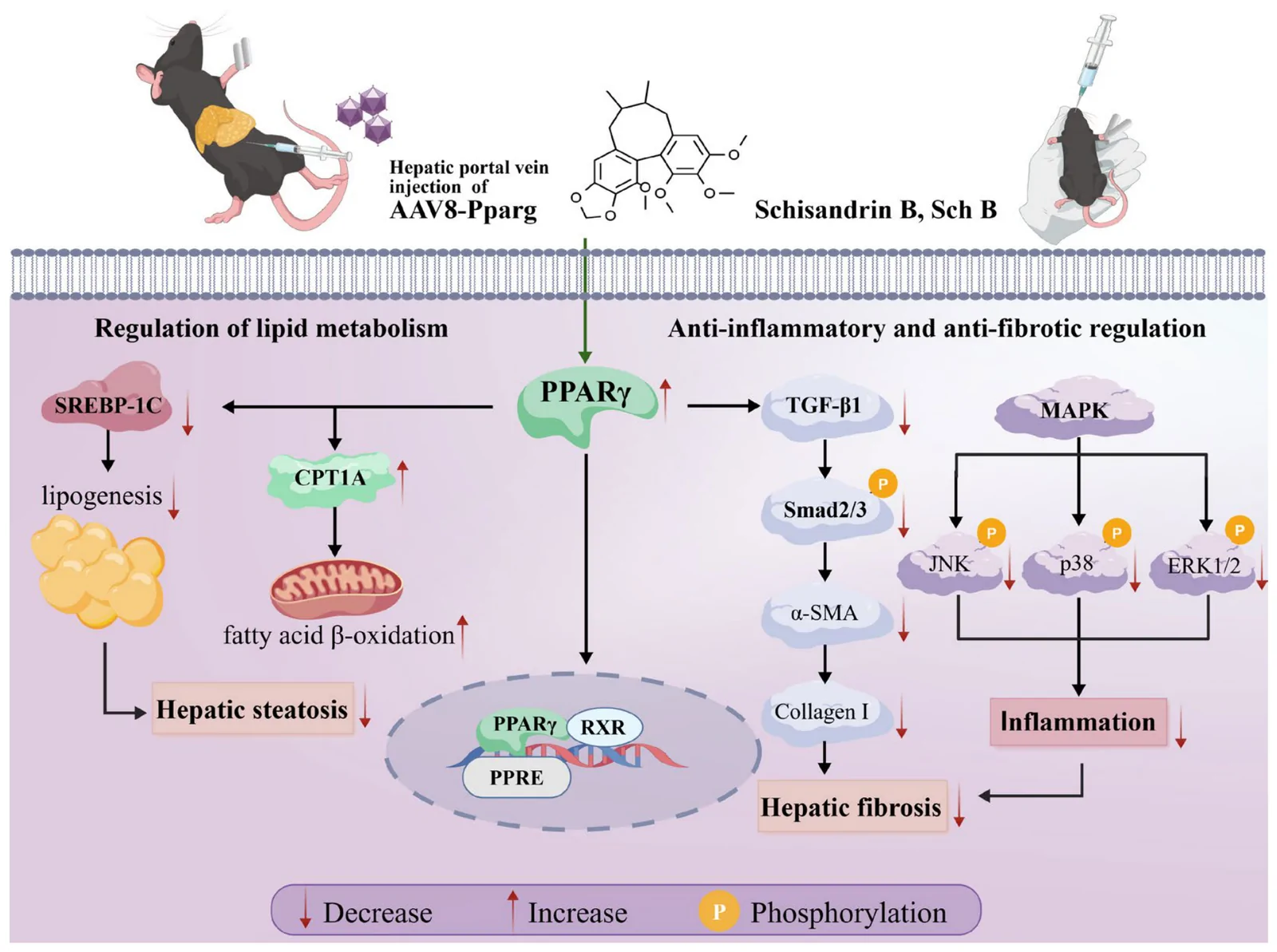
Mechanisms through which Sch B ameliorates MASLD in mice.

In summary, this study suggests that downregulation of PPARγ expression is an important molecular event driving the progression of MASLD. PPARγ deficiency can trigger an abnormal phosphorylation of the MAPK signaling cascade, leading to an imbalance in the expression of key lipid metabolism proteins such as SREBP-1c and CPT1A, promoting the release of TNF-α, IL-6, and IL-1β, and activating the TGF-β/Smad pro-fibrotic pathway, ultimately resulting in excessive lipid deposition, inflammatory infiltration, and fibrosis in liver cells. On the contrary, the overexpression of PPARγ and Sch B intervention can significantly restore PPARγ levels, inhibit the phosphorylation activation of the MAPK pathway, restore the steady-state expression of lipid metabolism-related proteins, reduce the production of pro-inflammatory factors, and block the transmission of TGF-β/Smad signals, thereby comprehensively alleviating liver lipid disorder, inflammatory damage, and fibrosis degree to play a multi-level protective role in MASLD (Figure 6). This study reveals the molecular mechanism of Sch B intervening in MASLD by regulating the PPARγ-MAPK signaling axis, verifying that PPARγ may be a potential therapeutic target and Sch B may be a candidate natural drug, which may provide a reliable experimental model and theoretical support for subsequent research.

The high-fat MCD diet induces body weight loss in mice, which represents an inherent limitation of this model, as it fails to capture the pathological features of obesity and systemic insulin resistance observed in patients with MASLD. Compared with the HFD and Western-diet models, which better capture systemic metabolic disturbances, the modified high-fat MCD model adopted in the present study is more suitable for investigating pathological changes specific to hepatic steatosis: inflammation and fibrosis. Meanwhile, several limitations of this study should be acknowledged. Only male mice were used in the current experiments, whereas the risk of MASLD is elevated in postmenopausal females; future studies using female animals would strengthen the clinical relevance of our findings. In addition, validation using PPARγ-knockout mice was not performed in this work. Therefore, it cannot be definitively concluded that the hepatoprotective effects of Sch B are specifically mediated via the PPARγ pathway, and further experiments are required to consolidate the mechanistic evidence.

## 4. Materials and Methods

### 4.1. Experimental Drugs and Reagents

Schisandrin B (purity > 99.00%) (Chengdu Pufide Biotechnology Co., Ltd., Chengdu, China); regular basal feed for mice (Changchun Yisi Experimental Animal Technology Co., Ltd., Changchun, China); high-fat methionine- and choline-deficient (MCD) model diet (TP36225MCD, Nantong Trofi Feed Technology Co., Ltd., Jiangsu, China); adeno-associated virus (AAV, Shanghai Jikai Gene Technology Co., Ltd., Shanghai, China); alanine aminotransferase (ALT), aspartate aminotransferase (AST), and total triglyceride (TG) detection kits (Nanjing Jiancheng Bioengineering Institute, Nanjing, China); interleukin-6 (IL-6), tumor necrosis factor alpha (TNF-α), and interleukin-1β (IL-1β) detection kits (Shanghai Enzyme linked Biotechnology Co., Ltd., Shanghai, China). Improved Oil Red O staining kit (Shanghai Biyun Tian Biotechnology Co., Ltd., Shanghai, China); hematoxylin and eosin (H&E) staining kit and Masson trichrome staining kit (Beijing Soleibao Technology Co., Ltd., Beijing, China); ECL color developer (Meilunbio^®^, Dalian, China); BCA protein assay kit, ammonium persulfate, 30% acrylamide, TEMED, 5 × SDS loading buffer, glycine, and HCl Tris (Beijing Dingguo Changsheng Biotechnology Co., Ltd., Beijing, China); skim milk powder (BD Company, Sparks, MD, USA). Antibody PPARγ, CPT1A, TGF-β1, Smad2/3, α-SMA, collagen I, JNK, P-JNK, p38, P-p38, ERK1/2, P-ERK1/2, and GAPDH (Nature Biosciences Co., Ltd., Zhejiang, China); SREBP-1c and P-Smad2/3 (Affinity Biosciences Co., Ltd., Jiangsu, China); HRP (ABclonal Co., Ltd., Wuhan, China).

### 4.2. Experimental Animals

Six-to-eight-week-old SPF male C57BL/6 mice were purchased from Changchun Yisi Experimental Animal Technology Co., Ltd. (SCXK (Ji) 2023-0002), Changchun, China. The temperature of the mouse feeding environment was 22–24 °C, the humidity was 55–60%, and the day and night alternated every 12 h. The animal experiments in this study were approved by the Beihua University Laboratory Animal Ethics Committee (BULAEC-2025093030), and the animal handling procedures were strictly carried out in accordance with the “Guidelines for the Care and Use of Laboratory Animals”.

Fifty C57BL/6 male mice were adaptively fed for one week and then randomly divided into 5 groups (*n* = 10), including a blank control (CON) group, MASLD model (MOD) group, Schisandrin B (Sch B 8 mg/kg) group, OE (sodium carboxymethyl cellulose, CMC-Na) group, and NC-OE (CMC-Na) group. The mice in the different groups were raised according to the corresponding feeding programs. The mice in the CON group were fed with regular basal diet, while those in the other four groups were fed with the high-fat MCD diet to establish the MASLD model. Starting from the 4th week, the mice in the different groups were intervened by injecting the corresponding intervention AAV solutions into the portal vein: mice in the OE (CMC-Na) group were injected with PPARγ-overexpression AAV (AAV8-Pparg), and those in the NC-OE (CMC-Na) group were injected with the PPARγ-overexpression-negative control AAV (AAV8-CON665). From the 5th week, mice in the Sch B 8 mg/kg group were orally administered with an equal volume of Sch B, while those in the other groups were orally administered with an equal volume of 0.5% carboxymethyl cellulose sodium (CMC-Na) solution as a solvent control. The mice were administered once a day, and the administration volume was dynamically adjusted according to the body weight of the mice to ensure accurate dosing. During the experiment, each group of mice was free to eat food and drink water, and their weight was measured and recorded at regular intervals every week. The growth of the mice was dynamically monitored.

**Figure 7:**
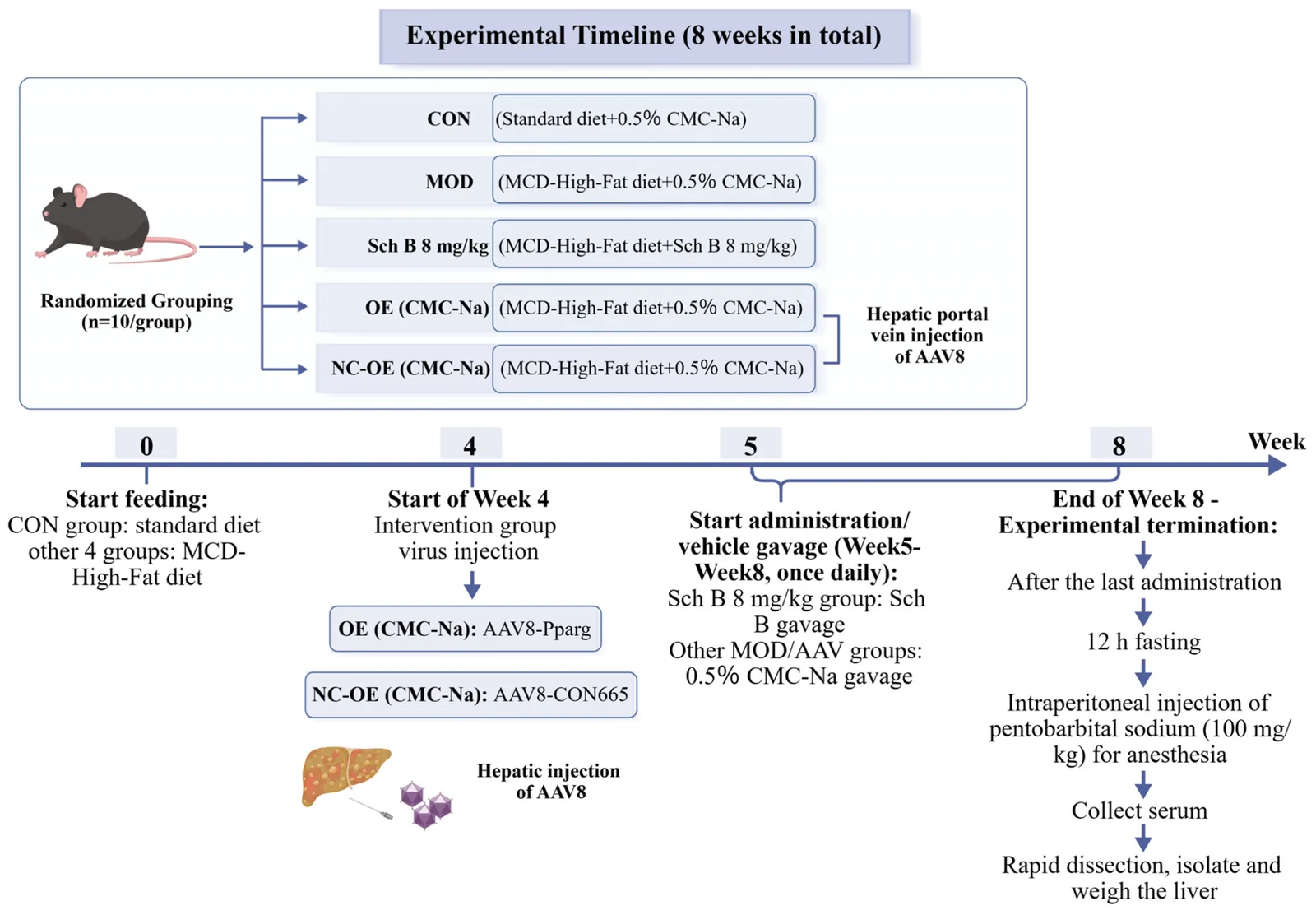
Animal grouping administration and experimental process.

After the end of the 8th administration, mice were fasted for 12 h (free to drink water) and anesthetized by intraperitoneal injection of pentobarbital sodium (100 mg/kg). Serum samples were collected after anesthesia, then the mice were immediately dissected and their liver tissue was isolated and removed. The serum samples and some liver tissue were immediately cryopreserved in a −80 °C ultra-low-temperature freezer for subsequent biochemical index detection, molecular mechanism analysis, etc. The remaining tissue was kept for pathological section preparation and related testing (Figure 7).

### 4.3. Injection of AAV Suspension

An ultra-clean work bench was pre-disinfected. The abdominal fur of the mouse was removed, and then the mouse was fixed in a supine position on a wooden board and anesthetized with isoflurane in continuous inhalation. Subsequently, the abdominal cavity of the mouse was opened and the intestines were gently pulled, then the needle of a syringe was slowly punctured into the hepatic portal vein at a very small angle and 100 μL of AAV suspension was injected into the portal vein at a constant speed, and the puncture site was pressed for more than 6 min to stop bleeding. The abdominal incision was sutured with absorbable sutures layer by layer. After surgery, the mice were treated with fluid replacement by injecting the fluid via subcutaneous injection, then raised in a separate cage for resuscitation and given penicillin potassium in intraperitoneal injection continuously for 3 days to prevent infection.

### 4.4. Determination of AST, ALT, TG, TNF-α, IL-1β, and IL-6

Serum AST, ALT, and TG levels were detected using the reagent kits. Inflammatory factors TNF-α, IL-1β, and IL-6 in the liver tissue of the mice were detected by ELISA.

### 4.5. Histopathological Examination

The liver tissue of the mice was fixed in a 4% paraformaldehyde solution, embed in paraffin, and stained with hematoxylin and eosin and Masson. The sections were mounted with neutral gum, and examined and photographed under a fluorescence inverted microscope (Nikon Corporation, Kanagawa, Japan). The slices were OCT-embedded and prepared into frozen sections, and stained with Oil Red O for detecting lipid deposition. After image acquisition, the Image J (Version 1.54g, National Institutes of Health, Bethesda, MD, USA) image analysis software was used for visualization analysis.

### 4.6. Western Blotting

The liver tissue samples were routinely prepared. The proteins were separated by sodium dodecyl sulfate polyacrylamide gel electrophoresis and transferred onto PVDF membranes, and skimmed milk powders were used for blocking. The primary antibodies of PPARγ (1:2000), SREBP-1c (1:2000), CPT1A (1:2000), TGF-β1 (1:2000), Smad2/3 (1:2000), P-Smad2/3 (1:2000), α-SMA (1:2000), collagen I (1:2000), JNK (1:2000), P-JNK (1:2000), p38 (1:2000), P-p38 (1:2000), ERK1/2 (1:2000), P-ERK1/2 (1:2000), and GAPDH (1:10,000) were added onto the membranes to incubate at 4 °C overnight. The next day, the secondary antibody HRP (1:50,000) was incubated with membranes for two hours, and the membranes were developed and photographed. The grayscale values of bands were measured using the Image J image analysis software, and the ratio of each grayscale value to the GAPDH grayscale value was taken as the relative expression level of the protein.

### 4.7. Statistical Analysis

All data were statistically analyzed using SigmaPlot 12.5 (Version 12.5, Systat Software, San Jose, CA, USA). The results were expressed as mean ± standard error (mean ± S.E.). For body-weight data, two-way ANOVA with repeated measures was adopted, with treatment group set as the between-subjects factor and week as the within-subjects factor. Greenhouse–Geisser correction was applied when the sphericity assumption was violated. Simple effect analysis with Bonferroni correction was used for pairwise comparisons at each time point. For other multiple-group comparisons, one-way ANOVA was performed, followed by Tukey’s post hoc multiple comparisons test. P < 0.05 was considered statistically significant. GraphPad Prism 9.5.0 (Version 9.5.0, GraphPad Software, Boston, MA, USA) and Generic Diagramming Platform (https://biogdp.com/, accessed on 1 July 2026) were used for image drawing [27]. The grayscale values of the WB bands were analyzed using the Image J software.

## 5. Conclusions

In this study, a mouse MASLD model was successfully established by giving male C57BL/6 mice a high-fat MCD diet, and the protective effect of Schisandrin B (Sch B) against MASLD by activating PPARγ and its mechanisms were investigated. The results indicate that PPARγ is significantly downregulated in MASLD and the downregulation is closely correlated with lipid deposition, inflammatory infiltration, and fibrosis severity. Both PPARγ overexpression and Sch B intervention can significantly alleviate MASLD. PPARγ may play a protective role against MASLD by regulating the MAPK pathway, while Sch B may play an anti-MASLD role by activating the PPARγ-MAPK axis, and its hepatoprotective, lipid-regulating, anti-inflammatory, and antifibrotic properties are all dependent on the regulation of the PPARγ-MAPK pathway. PPARγ may be a potential therapeutic target for MASLD, and Sch B may be expected to be a natural candidate drug targeting PPARγ.

## Data Availability

The original contributions presented in this study are included in the article. Further inquiries can be directed to the corresponding author.

## Abbreviations

The following abbreviations are used in this manuscript:

Sch B	Schisandrin B
MASLD	Metabolic dysfunction-associated steatotic liver disease
MASH	Metabolic dysfunction-associated steatohepatitis
PPPAγ	Peroxisome proliferator activated receptor gamma
CMC-Na	Sodium carboxymethyl cellulose
MCD	Methionine–choline deficient
OE	Overexpression
NC-OE	Negative control of overexpression
AAV	Adeno-associated virus
TGs	Triglycerides
ALT	Alanine aminotransferase
AST	Aspartate aminotransferase
H&E	Hematoxylin and eosin
SREBP-1c	Sterol regulatory element-binding protein 1c
CPT1A	Carnitine palmitoyltransferase 1A
TGF-β1	Transforming growth factor β1
α-SMA	α-smooth muscle actin
Collagen I	Collagen type I
Smad2/3	Smad family members 2/3
MAPK	Mitogen-activated protein kinase
JNK	c-Jun N-terminal kinase
P38	p38 mitogen-activated protein kinase
ERK1/2	Extracellular signal-regulated kinase 1/2
KEGG	Kyoto Encyclopedia of Genes and Genomes
